# A potential *cis*-regulatory element regulates differential expression of long wavelength-sensitive opsins in zebrafish

**DOI:** 10.1038/s41598-026-47667-4

**Published:** 2026-04-11

**Authors:** Lindsey M. Barrett, Robert D. Mackin, Ryuichi Ashino, Idris Korol, Benjamin M. Ting, Henry D. Pals, Shoji Kawamura, Deborah L. Stenkamp

**Affiliations:** 1 Department of Biological Sciences, University of Idaho, Moscow, ID USA; 2 Department of Integrated Biosciences, Graduate School of Frontier Sciences, The University of Tokyo, Kashiwa, 277-8562 Chiba Japan

**Keywords:** Cone photoreceptor, Opsin, Zebrafish, Thyroid hormone, Development, Cis-regulation, Developmental biology, Genetics, Molecular biology

## Abstract

Human long and medium wavelength-sensitive (LWS/MWS) opsins arose from a tandem duplication event resulting in opsins with divergent spectral sensitivities. Zebrafish independently underwent tandem duplication of their lws opsin, resulting in two spectrally divergent genes, lws1/lws2. Thyroid hormone (TH) differentially regulates these opsins; increased TH causes an increase in lws1 expression at the expense of lws2 (“opsin switching”). Here we investigate a cis-regulatory sequence that may mediate the response to TH using promoter-reporter analysis in transgenic zebrafish. We show that the 2.6-kb region upstream of lws1 combined with the 1.8-kb intergenic region are sufficient for typical lws1/2 expression and regulation by TH. We identify the 0.6-kb region upstream of lws1, as the likely region of TH-responsive cis-regulatory elements and use genomic prediction tools to uncover a putative lws regulatory element (LRE), in this 0.6-kb region. When we disrupt the LRE, we find that in larvae, lws1 reporter is prematurely expressed and does not respond to TH, and lws2 reporter increases in response to TH. In juveniles and adults, however, the patterning of lws1/2 reporters largely resembles stereotypical lws1/2 expression. Therefore, the LRE appears to be involved in differential expression of lws opsins and their response to TH during development.

**Supplementary Information:**

The online version contains supplementary material available at 10.1038/s41598-026-47667-4.

## Introduction

 Gene duplication is a major mechanism for the emergence of novel genes during evolution. Most often, duplicated genes are functionally redundant and undergo pseudogenization^1,2^. More rarely, genes will functionally diverge via subfunctionalization, where the daughter gene acquires a distinctive function related to the original gene, or neofunctionalization, where the daughter gene develops a different function entirely^1,2^. The genes encoding human long wavelength-sensitive (LWS; red) and medium wavelength sensitive (MWS; green) opsins underwent a tandem duplication head-to-tail on the X chromosome, which resulted in two spectrally divergent opsin genes, *LWS* and *MWS*^3^ under the control of an upstream regulatory region, the locus control region (LCR)^4^. The regulation of this locus is a topic of great interest, as LWS/MWS cones are implicated in color blindness^5,6^, X-linked achromatopsia^7^, X-linked cone dystrophies^8^, and some forms of blue cone monochromacy^9,10^. The regulation of this locus has previously been described as stochastic, where the LCR associates with either *LWS* or *MWS* and this determines the permanent fate of the photoreceptor^4^. However, this model does not explain the regionally specific spatiotemporal patterning of *LWS/MWS* expression in the retina, which suggests a transregulatory mechanism may be involved^11–13^. An alternative model is consistent with a transregulatory mechanism, involving spatiotemporal changes in LWS: MWS ratios during human fetal development, with potential functions for retinoic acid (RA) signaling noted in retinal organoids^12^.

Teleost fish, including zebrafish, also possess tandem duplications of their opsin genes, allowing them to be used as a model to study the genetic regulation of tandemly-duplicated opsin genes^14,15^. Zebrafish specifically have tandem replications of their *lws* and *rh2* opsin genes, with the *lws* opsin array having features similar to the human *LWS/MWS* locus^14,16^. The zebrafish *lws* locus contains a tandem duplication of the *lws* opsin gene on zebrafish chromosome 11, resulting in two spectrally divergent opsins, *lws1* and *lws2*^14^, which are regulated by an upstream enhancer region, the *lws* activating region (LAR)^17^. In larvae, *lws2* is expressed as early as 40 h post fertilization (hpf) and is expressed throughout the larval retina, whereas *lws1* is not expressed until days later, typically around 4–6 days post fertilization (dpf), in a localized ventral patch^18^. As the fish grows into juvenile and adulthood stages, *lws1* is restricted to the peripheral retina, concentrated ventrally and nasally, and *lws2* is expressed in the central and dorsal retina. We have previously shown that retinoic acid (RA) and thyroid hormone (TH) are essential regulators of *lws1/2* expression^19–23^. When treated with exogenous RA or TH, *lws1* expression increases, at the expense of *lws2* expression^19–22^. This “opsin switching” can be visualized through live imaging of transgenic zebrafish larvae which report *lws1* and *lws2* expression with fluorescent reporters, where *lws2* reporter-expressing cones switch to *lws1* reporter expression in response to exogenous TH^21^. Disrupting TH signaling increases *lws2* expression, and completely abolishes *lws1* expression, except for the area of the ventral retina known to be a domain and target of RA signaling^21,22^. *lws1* and *lws2* expression can be rescued from disrupted TH signaling through supplementation with exogenous TH, further demonstrating that the expression of *lws1* and *lws2* is plastic throughout the life of the fish^20–22^.

TH is a nuclear signaling molecule that associates with TH receptors (TRs), which bind sequences of DNA called TH response elements (TREs) in accessible euchromatin to recruit factors that affect gene expression^24–26^. TRs can homodimerize or heterodimerize with other members of the nuclear hormone receptor family, including vitamin D receptors, retinoic acid receptors (RARs), and retinoid X receptors (RXRs)^27–29^. There are multiple types of TREs which each preferentially bind different homodimer and heterodimer combinations and allow for recruitment of different cofactors depending on the liganded state of the receptor^30^. Most of the work done on TREs has been performed using human receptors, and three types of canonical TREs have been discovered based on the human canonical half-site sequence AGGTCA: direct repeats with 4-bp spacer (DR4), inverted repeats with a 6-bp spacer (IR6), or palindromic repeats with no spacer (ER0) (Suppl. Figure 1)^29–33^. Distinct organisms may have variable half-site sequences or lengths of spacer sequences, but many have motifs similar to the human canonical TREs^34,35^. While homodimers and heterodimers have the capacity to bind each type of TRE, different combinations of receptors preferentially bind different TRE arrangements^30^. For example, heterodimers tend to preferentially bind DR4 elements^29,30^, while homodimers of human and chick TRβ isoforms have been shown to preferentially bind inverted repeat elements^35–38^. Though there has not yet been direct evidence of TR binding at the zebrafish *lws* locus, a splice variant of one of the vertebrate TRs, *trβ2*, is expressed in cones and is known to be essential for LWS cone fate and opsin expression^23,39–41^. Further, a recent ChAP-Seq approach using mouse retina identified numerous TR binding sites; the most frequent consensus motif at these sites was a DR4 (direct repeat of AGGTCA-like sequences with four bases intervening)^42^. Additionally, we have recently collected evidence consistent with the hypothesis that liganded Trβ2 upregulates *lws1* and unliganded Trβ2 tends to promote *lws2* expression^23^.

Tsujimura et al., 2010 investigated the elements necessary for *lws1/2* expression and led to the discovery of the LAR through the microinjection of expression constructs containing regulatory regions of the *lws* locus driving expression of fluorescent reporters^17^. Through the study of these constructs, they found that the 1.8 kb region upstream of *lws2* is not sufficient to allow for the expression of *lws2*, but when the LAR is placed in front of the 1.8 kb region upstream of *lws2*, reporter expression was observed in all LWS cones in the retina^17^. This suggests that the LAR is necessary for *lws2* expression but does not contain the elements necessary to restrict *lws2 +* cones to their proper domain. However, *lws1* and *lws2* reporter expression displayed proper patterning in a double reporter, which was made using the 2.6-kb region upstream of *lws1* (including the LAR) driving expression of GFP and the 1.8-kb region of *lws2* driving expression of RFP^17^. Taken together, this suggests that there must be an additional element(s), other than the LAR, in the region upstream of *lws1* necessary for normal patterning of *lws1* vs. *lws2*.

Therefore, in the present study, we aimed to identify those specific elements(s). We show that the 0.6 kb region upstream of *lws1*, between the LAR and *lws1*, is likely necessary for the expression of *lws1* and the normal response of *lws2* to TH in an in vivo promoter-reporter system. This suggests that the 0.6-kb region upstream of *lws1* is likely the location of *cis*-regulatory elements in the *lws* locus that regulate differential expression. We investigated this region further using genomic prediction tools^21,43–45^ to find predicted TREs in the 0.6 kb region upstream of *lws1* and found a 35-bp element which we refer to as the *lws* regulatory element (LRE). This element contains one canonical AGGTCA half-site, a potential inverted repeat-like sequence with a 13-bp spacer, a potential direct repeat-like sequence with a 9-bp spacer, and a potential direct repeat-like sequence with a 4-bp spacer (DR4) (Suppl. Figure 1). We created a promoter-reporter construct containing a disruption of the LRE and found that an intact LRE appears to be necessary for normal patterning of *lws1/2* reporters and responsiveness to TH at embryonic/larval timepoints, but another regulatory mechanism may compensate for the loss of the LRE later in the life of the fish. The discovery of this potential *cis*-regulatory element will lead to a greater understanding of the regulation of tandemly replicated opsins.

## Results

### *Cis*-regulatory elements needed within a promoter-reporter system for replication of endogenous patterns of *lws1/2* expression and the response to thyroid hormone (TH) reside in the 2.6-kb upstream of *lws1*

**Fig. 1:**
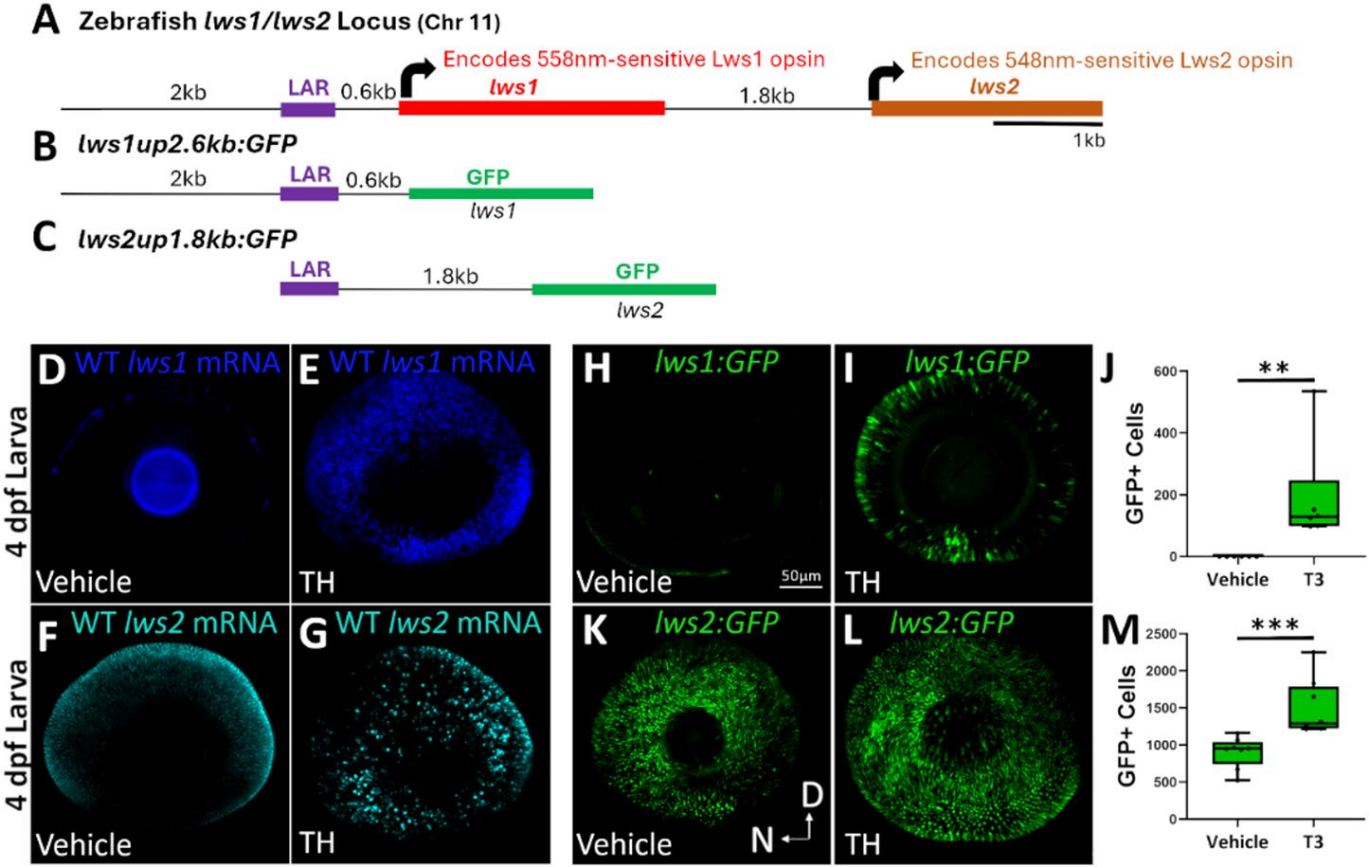
In a promoter-reporter system, the 2.6-kb region upstream of *lws1* is sufficient for the T3 regulation of *lws1* reporter, but the 1.8-kb region upstream of *lws2* is not sufficient for T3 downregulation of *lws2* reporter. (**A**-**C**) Schematic representations of (**A**) the native *lws* locus, which contains the *lws* activating region (LAR) and tandemly replicated *lws1* and *lws2* genes, (**B**) the *lws1up2.6 kb: GFP* reporter construct, and (**C**) the *LAR: lws2up1.8 kb: GFP* reporter construct. (**D**-**G**) Whole-mounted 4-dpf WT eyes labeled with fluorescent HCR in situ probes for *lws1* mRNA (**D**,**E**) and *lws2* mRNA (**F**,**G**) visualized using confocal microscopy of (**D**,**F**) DMSO-treated (vehicle) or (**E**,**G**) T3-treated larvae. (**H**,**I**) Whole-mounted 4-dpf *lws1up2.6 kb: GFP* eyes visualized using confocal microscopy of (**H**) DMSO-treated (vehicle) or (**I**) T3-treated larvae. (**J**) Numbers of GFP+ cones for DMSO vs. T3 treated *lws1up2.6 kb: GFP* (*n* = 6 for each; *p* = 0.00512). (**K**,**L**) Whole-mounted 4-dpf *LAR: lws2up1.8 kb: GFP* eyes of larvae treated with (K) DMSO or (**L**) T3. (M) Numbers of GFP+ cones for DMSO vs. T3 treated *LAR: lws2up1.8 kb: GFP* (*n* = 8 for each; *p* = 0.00094). *p* values were calculated using the Mann-Whitney U test; **p < 0.01, ***p < 0.001. D = dorsal; N = nasal.

We utilized two stable transgenic lines to test the hypothesis that the *cis*-regulatory elements needed for TH-mediated regulation of the zebrafish *lws* locus (Fig. 1A-C) are the same as those needed for the establishment of endogenous spatiotemporal *lws1/2* expression patterns (Fig. 1D-G). The first, *lws1up2.6 kb: GFP* (Fig. 1B), reports *lws1* expression with GFP. This line displays a pattern of GFP expression consistent with endogenous *lws1* expression, with little to no reporter expression at 4 days post fertilization (dpf) (Fig. 1H)^17^. When treated from 2 to 4 dpf with 100 nM triiodothyronine (T3), a treatment that is known to increase *lws1* expression at the expense of *lws2* expression^19,21^, an increase in GFP expression was observed in comparison to DMSO-treated controls (Fig. 1H, I). When numbers of GFP+ cones were counted, there was a significant increase in GFP+ cells counted in T3-treated larvae compared to DMSO-treated controls (Fig. 1J), which is consistent with previous data reported in larvae from a separate transgenic line, *lws: PAC(H)*, in which the transgene consists of ~ 110 kb of zebrafish chromosome 11 with GFP reporting *lws1* and RFP reporting *lws2*^17,21^. Together, these findings indicate that the proximal 2.6-kb region upstream of *lws1* contains elements sufficient for TH-mediated regulation of *lws1* reporter.

The second transgenic line, *LAR: lws2up1.8 kb: GFP* (Fig. 1C), should report *lws2* expression with GFP, yet GFP is expressed within all LWS cones of the adult retina^17^. These findings suggest that the LAR and 1.8-kb intergenic region together do not contain the elements necessary to restrict expression of *lws2* to the central retina. Larvae treated from 2 to 4 dpf with 100 nM T3 did not show the expected reduction of GFP+ cones (reporting *lws2*) in comparison with controls (Fig. 1K, L)^21^, but rather an increase in GFP+ cells (Fig. 1M). This suggests that the LAR and 1.8-kb intergenic region together do not contain the elements necessary to reduce *lws2* reporter expression in response to T3. However, they may contain an element that promotes expression of *lws2* reporter in response to T3, at least in the absence of other elements in the upstream region of the locus. Together, these two stable transgenic lines suggest that the *cis*-regulatory elements for regulating *lws1/2* reporter expression and mediating the response to TH likely reside in the 2.6-kb region immediately upstream of *lws1*.

### The 2.6-kb region upstream of *lws1* combined with the 1.8-kb region upstream of *lws2* are sufficient to faithfully report *lws1* and *lws2* endogenous expression patterns

**Fig. 2:**
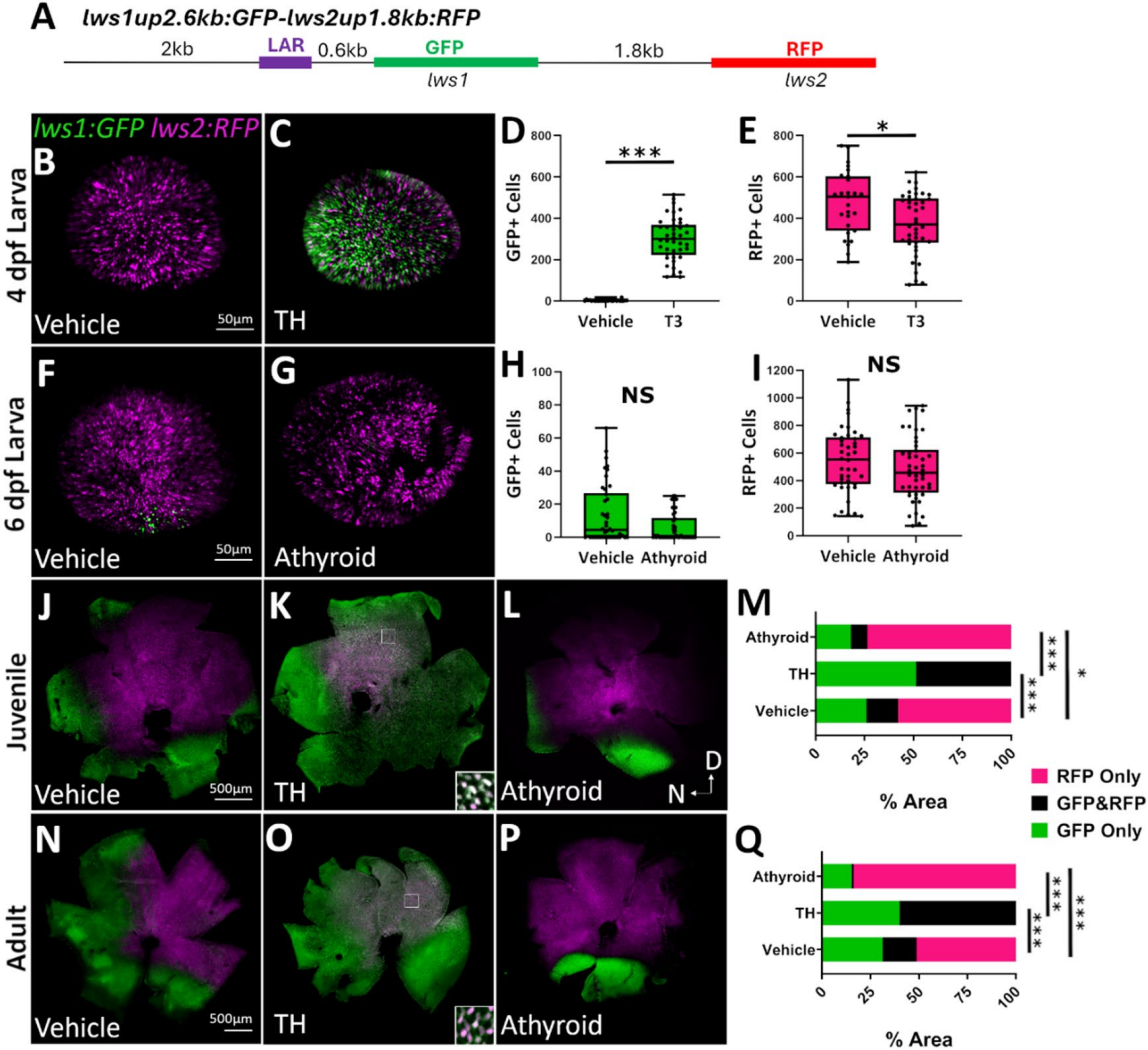
The 2.6-kb region upstream of *lws1* combined with the 1.8-kb region upstream of *lws2* is sufficient for the endogenous patterning and regulation by TH of *lws1/2* reporter. (**A**) Schematic representation of the *lws1up2.6 kb: GFP-lws2up1.8 kb: RFP* (*lws1:GFP-lws2:RFP*) reporter construct. (**B**,**C**) Whole mounted 4-dpf *lws1:GFP-lws2:RFP* eyes visualized using confocal microscopy of (B) DMSO-treated (vehicle) or (**C**) T3-treated larvae. (**D**,**E**) Numbers of (**D**) GFP+ (*p* = 2.65E-12) and (**E**) RFP+ (*p* = 0.0144) cones for DMSO (*n* = 18) vs. T3 treated (*n* = 27) larvae. (**F**,**G**) Whole mounted 6-dpf *lws1:GFP-lws2:RFP* eyes visualized using confocal microscopy of larvae treated with (**F**) DMSO or (**G**) Mtz from 2–4 dpf to ablate the thyroid. (**H**,**I**) Numbers of (**H**) GFP+ (*p* = 0.0844) and (**I**) RFP+ (*p* = 0.0549) cones for DMSO (*n* = 18) vs. Mtz treated (*n* = 27) larvae. *p* values were calculated using the Mann-Whitney U test for D, E, and H, and a Student’s t-test for I; *p < 0.05, ***p < 0.001. (**J**-**L**) Whole mounted *lws1:GFP-lws2:RFP* retinas visualized using confocal microscopy of (**J**) vehicle-treated (NaOH or DMSO; *n* = 10), (**L**) T4-treated (*n* = 5), or (**L**) athyroid (*n* = 5) juveniles. (**M**) Percent area analysis of regions of GFP only, RFP only, and GFP&RFP coexpressed/interspersed for juvenile retinas. Fisher’s Exact test was used to test for overall differences between treatment groups: Vehicle vs. T4 *p* = 0; Vehicle vs. Athyroid *p* = 0.0443; T4 vs. Athyroid *p* = 0 with 3 × 2 contingency tables; *p < 0.05, ***p < 0.001. (N-P) Whole mounted *lws1:GFP-lws2:RFP* retinas visualized using confocal microscopy of adults treated with (**N**) vehicle (*n* = 9), (**O**) T4 (*n* = 6), or (**P**) athyroid (*n* = 5). (**Q**) Percent area analysis of regions of GFP only, RFP only, and GFP & RFP coexpressed/interspersed for adult retinas. Fisher’s Exact test was used to test for overall differences between treatment groups: *p* = 0 for all with 3 × 2 contingency tables; ***p < 0.001. D = dorsal; N = nasal. Regions bounded by squares in K and O show location of enlarged areas (insets) demonstrating coexpression.

Previously, *lws: PAC(H)* has been used to faithfully report *lws1/2* expression^17,21^. We aimed to design a *lws1/2* reporter that has far less sequence to more specifically localize the regulatory elements necessary for the spatiotemporal patterning and TH-regulation of *lws1/2*. We created a transgenic line, *lws1up2.6 kb: GFP-lws2up1.8 kb: RFP* (*lws1:GFP-lws2:RFP*), where the 2.6-kb region upstream of *lws1* drives expression of GFP as a reporter of *lws1* expression, and the 1.8-kb intergenic region upstream of *lws2* drives expression of RFP as a reporter of *lws2* expression (Fig. 2A)^17^. This line appears to faithfully report *lws1/2* expression throughout the life of the fish when compared to previous *lws: Pac(H)* reporter expression^17,19,21^ and hybridization chain reaction (HCR) in situ to detect native *lws1/2* mRNA^19,20,23^. In 4-dpf larvae, there is little to no *lws1* reporter present under endogenous TH conditions (Fig. 2B), but when treated from 2 to 4 dpf with 100 nM T3, an increase in GFP expression and a decrease in RFP expression was observed in comparison to DMSO-treated controls (Fig. 2B, C). When numbers of GFP + and RFP+ cones were counted (Fig. 2D, E), there was a significant increase in the number of GFP+ cells and a significant decrease in the number of RFP+ cells in response to TH treatment, which is consistent with previous *lws: Pac(H)*^17,19,21^ and HCR in situ^19,20,23^ data. At 6 dpf, there was an increase in *lws1* reporter positive cells in the ventral retina (Fig. 2F), consistent with previous reports in *lws: PAC(H)* larvae at this timepoint^21^. *lws1:GFP-lws2:RFP* fish were crossed with a transgenic line, *Tg(tg: nVenus-2a-nfnB)*^*wp.rt8*^, which allows for the chemical ablation of the thyroid using metronidazole (Mtz) treatment^46^. When the thyroid was ablated with Mtz treatment from 2 to 4 dpf and eyes were collected for reporter imaging at 6 dpf, the number of GFP+ cells appeared to decrease compared to DMSO controls (Fig. 2G). GFP + and RFP+ cell counts did not reveal a significant decrease in GFP+ cells, though the counts did trend downward in athyroid larvae (Fig. 2H, I). Athyroid larvae showed no difference in the number of RFP+ cells, similar to findings for *lws: PAC(H)* larvae^21^. These results suggest that *lws1:GFP-lws2:RFP* closely reports *lws1/2* expression at larval timepoints and in response to manipulations of TH levels.

**Fig. 3:**
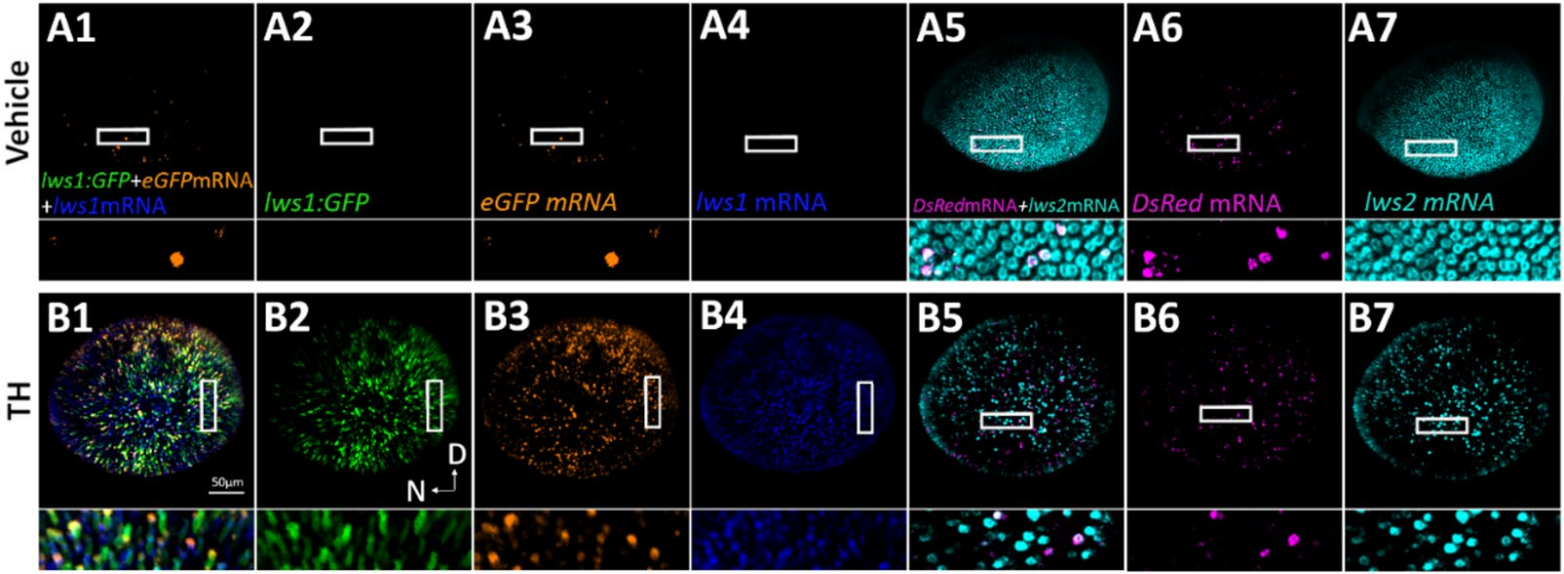
HCR in situ reveals overlapping expression pattern of GFP and RFP reporters with native *lws1/2* mRNA transcripts. **(A1-A6)** Whole mounted 4 dpf DMSO (vehicle)-treated *lws1:GFP-lws2:RFP* eye labeled with fluorescent HCR in situ probes for *eGFP* mRNA **(A1**,**A3)**, *lws1* mRNA **(A1**,**A4)**, *DsRed-Express* mRNA **(A5**,**A6)**, and *lws2* mRNA **(A5**,**A7)** visualized using confocal microscopy. **(B1-B6)** Whole mounted 4-dpf T3-treated *lws1:GFP-lws2:RFP* eye labeled with fluorescent HCR in situ probes for *eGFP* mRNA **(B1**,**B3)**, *lws1* mRNA **(B1**,**B4)**, *DsRed-Express* mRNA **(B5**,**B6)**, and *lws2* mRNA **(B5**,**B7)** visualized using confocal microscopy. D = dorsal; N = nasal. Rectangles show location of enlarged areas demonstrating coexpression.

In juveniles (1 month post-fertilization; mpf) and adults (3–6 mpf), the pattern of *lws1/2* reporter expression also appears to be consistent with *lws1/2* patterning observed in previous studies^20,21^, where *lws1* reporter expression is isolated to the ventral and nasal periphery and *lws2* reporter expression is located in the central and dorsal retina^18^(Fig. 2J, N). When juveniles (Fig. 2K) and adults (Fig. 2O) were treated with 386 nM thyroxine (T4) for 5 days, GFP expression increased throughout the entire retina. RFP expression decreased and was isolated to a small patch in the central dorsal retina that coexpressed GFP reporter. To assess the effect of athyroidy, *lws1:GFP-lws2:RFP* transgenics were crossed with *Tg(tg: nVenus-2a-nfnB)*^*wp.rt8*^ transgenics, treated with Mtz from 2 to 4 dpf to ablate the thyroid, and allowed to grow to juvenile or adult ages. Athyroid juveniles (Fig. 2L) and adults (Fig. 2P) displayed a reduction in GFP reporter expression, which was isolated to a ventral patch, which is likely the result of retinoic acid (RA) signaling known to be active in the ventral retina^21,22^. Regions of GFP+ only, RFP+ only, and GFP/RFP coexpression/interspersion were calculated as a percentage of the whole retina for juveniles (Fig. 2M) and adults (Fig. 2Q). When treated with TH, regions of exclusively RFP+ cells decreased from 51% to 0% and were replaced with regions of GFP/RFP coexpression/interspersion and exclusively GFP+ cells which increased from 18% to 60% and 31% to 40%, respectively. Athyroidy had the opposite effect, where areas of exclusively GFP+ cells and GFP/RFP coexpression/interspersion decreased from 31% to 18% and 18% to 0%, respectively, in favor of regions of exclusively RFP+ cells, which increased from 51% to 82%.

To further clarify that the *lws1:GFP-lws2:RFP* transgenic line closely reports native *lws1/2* expression, we performed fluorescent HCR in situ to label native *lws1* and *lws2* transcript, and transcript of the GFP and RFP reporters used in the transgenic construct, specifically *eGFP* and *dsRed-Express* (dsRed-Express is the “RFP”). The HCR images from 4-dpf larvae treated from 2 to 4 dpf with DMSO (Fig. 3A1-A7) or 100 nM T3 (Fig. 3B1-B7) reveal that native *lws1* mRNA colocalizes with GFP reporter protein expression and *eGFP* mRNA (Fig. 3A1-A4;B1-B4). Native *lws2* mRNA also appears to colocalize with *dsRed-Express* mRNA, though the *dsRed* mRNA+ profiles were not as numerous as the *lws2* mRNA+ profiles(Fig. 3A5-A7; B5-B7). It has been reported that *dsRed* mRNA levels can be “uncoupled” from dsRed fluorescent protein accumulation, likely due to variable mRNA turnover rates^47^. It is possible that this is occurring in the *lws1:GFP-lws2:RFP* transgenics as well. dsRed-Express protein is likely denatured by the HCR process, as it could not be visualized. When all the above results are combined, they suggest that the *lws1:GFP-lws2:RFP* transgenic line is a suitable reporter of *lws1/2* expression throughout the development of the zebrafish, and the 2.6-kb region upstream of *lws1* combined with the 1.8-kb region upstream of *lws2* have sufficient regulatory sequence to allow for the patterns of reporter expression consistent with normal expression of *lws1/2*.

### *Cis*-regulatory elements needed within a promoter-reporter system for replication of endogenous patterns of *lws1/2* expression and the response to TH likely reside in the 0.6-kb region immediately upstream of *lws1*

**Fig. 4:**
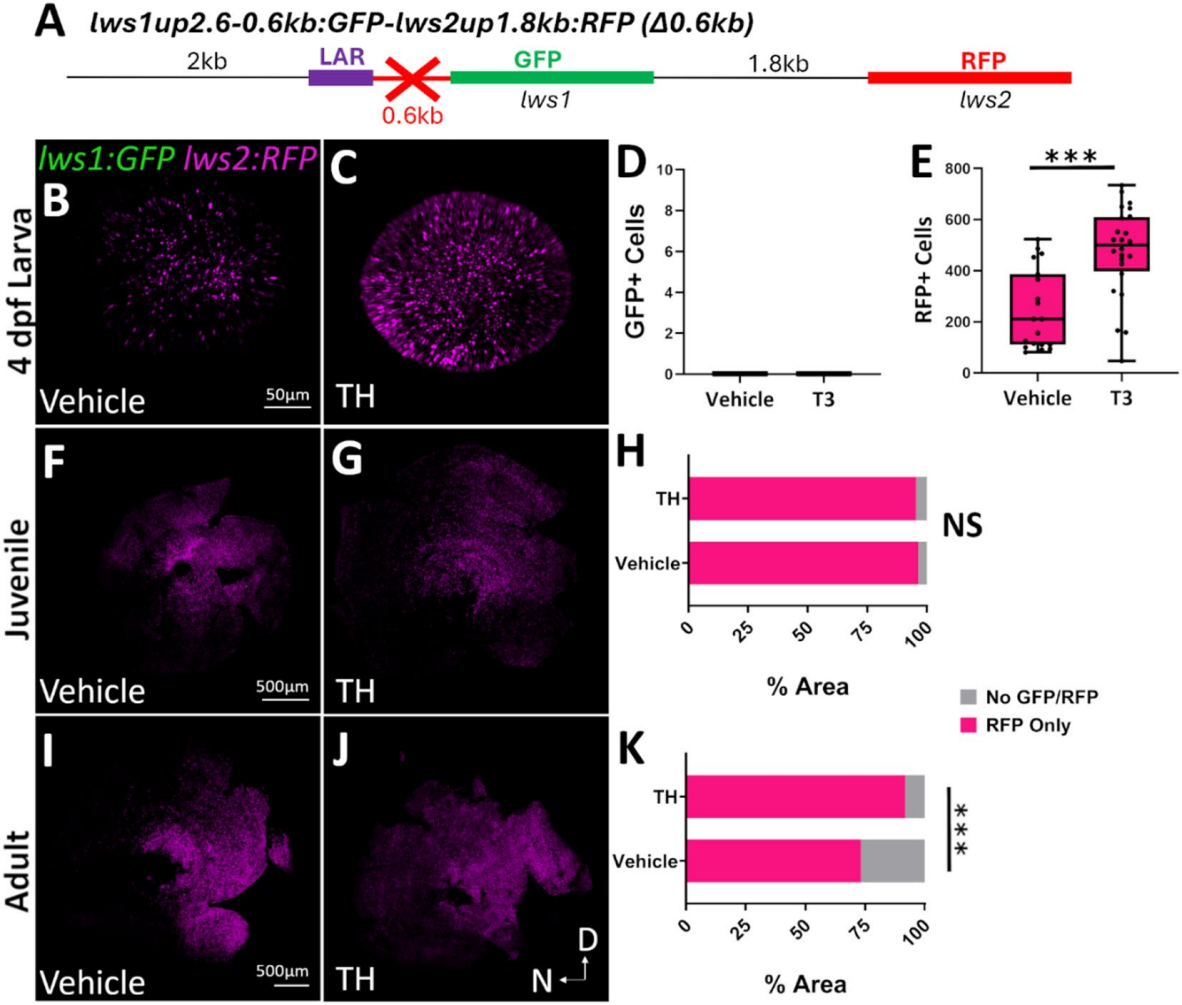
In a promoter-reporter system, the 0.6-kb region upstream of *lws1* is required for *lws1* reporter expression and the normal TH regulation of *lws1/2* reporters. **(A)** Schematic representations of the *lws1up2.6-0.6.6 kb: GFP*-*lws2up1.8 kb: RFP (Δ0.6kb)* reporter construct. **(B**,** C)** Whole mounted 4-dpf *Δ0.6kb* eyes visualized using confocal microscopy of **(B)** DMSO-treated (vehicle) or **(C)** T3-treated larvae. **(D**,** E)** Numbers of **(D)** GFP+ (*p* = 0) and **(E)** RFP+ (*p* = 0.000211) cones for DMSO (*n* = 11) vs. T3 treated (*n* = 13) larvae. *p* values were calculated using the Mann-Whitney U test. **(F**,** G)** Whole mounted *Δ0.6kb* retinas visualized using confocal microscopy of **(F)** NaOH-treated (vehicle; *n* = 4) or **(G)** T4-treated (*n* = 4) juveniles. **(H)** Percent area analysis of regions of GFP only, RFP only, GFP & RFP coexpressed/interspersed, or no GFP/RFP for juvenile retinas. Fisher’s Exact test was used to test for overall differences between treatment groups: *p* = 1 with a 2 × 2 contingency table. **(I**,** J)** Whole mounted *Δ0.6kb* retinas visualized using confocal microscopy of adults treated with **(I)** vehicle (*n* = 5) or **(J)** T4 (*n* = 6). **(K)** Percent area analysis of regions of GFP only, RFP only, GFP & RFP coexpressed/interspersed, and no GFP/RFP for adult retinas. Fisher’s Exact test was used to test for overall differences between treatment groups: *p* = 0.0006 with a 2 × 2 contingency table; ***p < 0.001. D = dorsal; N = nasal.

We hypothesized that the elements necessary for the spatiotemporal patterning of *lws1/2* and their regulation by TH may be in the 0.6 kb region upstream of *lws1*, because results using the *lws1up1.3 kb: GFP: lws2up1.8 kb: RFP* construct, which contains a deletion of the 2 kb region upstream of the LAR, found normal *lws1/2* reporter expression patterns and response to TH in transiently expressing transgenic larvae (Suppl. Figure 2). We created the *lws1up2.6-0.6.6 kb: GFP*-*lws2up1.8 kb: RFP (Δ0.6kb)* construct by deleting the 0.6-kb region upstream of *lws1* between the LAR and *lws1* from the *lws1:GFP-lws2:RFP* construct (Fig. 4A). The *Δ0.6kb* line displays *lws2* reporter expression throughout the retina, with no *lws1* reporter expression in larval (Fig. 4B), juvenile (Fig. 4F), or adult (Fig. 4I) timepoints, which suggests that the 0.6-kb region upstream of *lws1* is required for *lws1* reporter expression. When treated with 100 nM T3 from 2 to 4 dpf for larvae (Fig. 4C) and 386 nM T4 for 5 days for juveniles (Fig. 4G) and adults (Fig. 4J), *lws2* reporter expression appears to increase when *lws2* mRNA itself would normally decrease in response to TH. *lws1* reporter was still not expressed. These results were confirmed by GFP+ (Fig. 4D) and RFP+ (Fig. 4E) cell quantification for larval eyes and regional percentage calculations of GFP+ only, RFP+ only, GFP/RFP coexpression/interspersion, and no GFP/RFP + for juvenile (Fig. 4H) and adult (Fig. 4K) whole retinas. In larvae, the number of RFP+ cells significantly increased in response to TH, compared to DMSO controls. In juvenile retinas, no change in percent areas of exclusively RFP+ cells were observed (vehicle 96%, TH 95%). In adults, vehicle-treated retinas displayed regions of no GFP + or RFP+ cells, which encompassed 27% of the retina. When treated with TH, these areas of no GFP/RFP+ cells decreased to 8% and were filled by RFP+ cells, which increased from 73% to 92% in response to TH. This suggests that not only is the 0.6-kb region upstream of *lws1* required for *lws1* reporter expression, but it may also be necessary for the normal regulation of the *lws* reporters in response to TH. Therefore, the *cis*-regulatory element(s) necessary for the endogenous patterning and responsiveness to TH of *lws1/2*, likely reside in this 0.6-kb region.

### An element in the 0.6-kb region immediately upstream of *lws1* may be responsible for the suppression of *lws1* reporter and responsiveness of *lws1/2* reporter expression to TH in early development and for maintenance of *lws2* reporter expression later in development

**Fig. 5:**
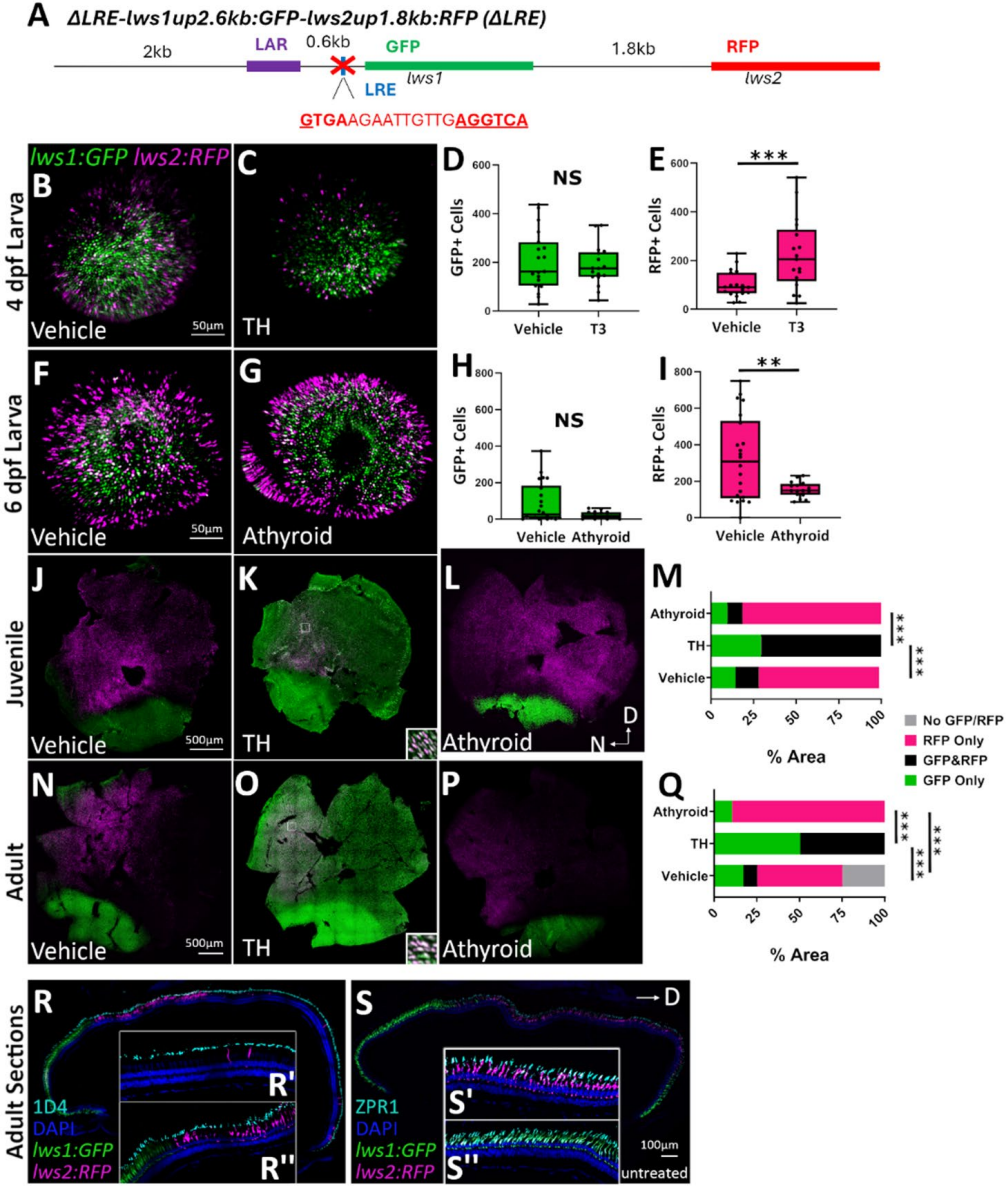
In a promoter-reporter system, the LRE may be involved in the suppression of *lws1* reporter and responsiveness of *lws1/*2 reporter expression to TH in early development, and for the maintenance of *lws2* reporter expression in adults. **(A)** Schematic representation of the *ΔLRE-lws1:GFP-lws2up1.8 kb: RFP* (*ΔLRE*) reporter construct. **(B**,** C)** Whole mounted 4-dpf *ΔLRE* eyes visualized using confocal microscopy of **(B)** DMSO-treated (vehicle) or **(C)** T3-treated larvae. **(D**,** E)** Numbers of **(D)** GFP+ (*p* = 0.283) and **(E)** RFP+ (*p* = 0.000747) cells for DMSO (*n* = 14) vs T3 treated (*n* = 13) larvae. The *p* values were calculated using a Student’s t-test; ***p < 0.001 **(F**,** G)** Whole mounted 6dpf *ΔLRE* eyes visualized using confocal microscopy of larvae treated with **(F)** DMSO or **(G)** Mtz from 2–4 dpf to ablate the thyroid. **(H**,** I)** Numbers of **(H)** GFP+ (*p* = 0.235) and **(I)** RFP+ (*p* = 0.00323) cones for DMSO (*n* = 12) vs Mtz treated (*n* = 11) larvae. The *p* values were calculated using the Mann-Whitney U test for H and a Student’s t-test for I; **p < 0.01. **(J-L)** Whole mounted *ΔLRE* retinas visualized using confocal microscopy of **(J)** vehicle-treated (NaOH or DMSO; *n* = 10), **(K)** T4-treated (*n* = 5), or **(L)** athyroid (*n* = 3) juveniles. **(M)** Percent area analysis of regions of GFP only, RFP only, and GFP & RFP coexpressed/interspersed for juvenile retinas. Fisher’s Exact test was used to test for overall differences between treatment groups: Vehicle vs T4 *p* = 0; Vehicle vs Athyroid *p* = 0.4344; T4 vs Athyroid *p* = 0 with 3 × 2 contingency tables; ***p < 0.001. **(N-P)** Whole mounted *ΔLRE* retinas visualized using confocal microscopy of adults treated with **(N)** vehicle (*n* = 17), **(O)** T4 (*n* = 8), or **(P)** athyroid (*n* = 3). **(Q)** Percent area analysis of regions of GFP only, RFP only, GFP & RFP coexpressed/interspersed, and no GFP/RFP for adult retinas. Fisher’s Exact test was used to test for overall differences between treatment groups: *p* = 0 for all with a 4 × 2 contingency table; ***p < 0.001. **(R-S)** Retinal cryosections of untreated adult retinas labeled with **(R)** 1D4 antibody to label all LWS cones (LWS1 and LWS2 cones) in zebrafish and **(S)** ZPR1 antibody which labels all double cones (LWS and RH2 cones) in zebrafish. **(R’**,**R’’)** Enhanced regions of 1D4 labeled retina showing **(R’)** *lws2* sparseness and **(R’’)** GFP and RFP reporter coexpression with 1D4 antibody label. **(S’-S’’)** Enhanced regions of ZPR1 labeled retina showing **(S’)** RFP and **(S’’)** GFP reporter coexpression with ZPR1 label. D = dorsal; N = nasal. Regions bounded by squares in K and O show location of enlarged areas (insets) demonstrating coexpression.

In order to find more specific elements that may be involved in the endogenous patterning and TH regulation of the *lws* locus, we used genomic prediction tools, MatInspector^43^ and Promo^44,45^, to find putative thyroid hormone response elements (TREs) and retinoid X response elements (RXREs) in the 0.6-kb region upstream of *lws1*^21,22^. Through this analysis, we identified a 35-bp region 0.2-kb upstream of *lws1*, which we refer to as the *lws* regulatory element (LRE). There are three well-described canonical human TREs, which include: direct repeats with a 4-bp spacer (DR4), inverted repeats with a 6-bp spacer, or palindromic repeats with no spacer using the canonical half-site sequence, AGGTCA (Suppl. Figure 1)^29–33^. The LRE contains one such canonical half-site, a potential inverted repeat-like sequence with a 13-bp spacer, a potential direct repeat-like sequence with a 9-bp spacer, and a potential DR4-like sequence, (some of which are overlapping) (Suppl. Figure 1). Zebrafish Trβ2 has been found to influence *lws1 vs. lws2* expression^23^ and in mice, may heterodimerize with RXRγ to decide cone fate^27,48^. If Trβ2 were to bind at the *lws* locus, whether it homodimerizes or heterodimerizes, the LRE may be a promising as a potential binding site. However, we must also emphasize that there is no evidence to date that TH exerts a direct effect upon a TR that occupies site(s) upon the *lws* locus in zebrafish. To investigate the involvement in the regulation of the *lws* locus of this element, we modified the *lws1:GFP-lws2:RFP* construct by disrupting 20 bp of the 35-bp region encompassing the LRE to make the construct *ΔLRE-lws1up2.6 kb: GFPlws2up1.8 kb: RFP (ΔLRE)* (Fig. 5A; Suppl. Figure 3).

We found a fascinating pattern of *lws1/2* reporter expression in larval zebrafish, with premature and variable *lws1* reporter expression ectopically localized in the central retina and *lws2* reporter expression throughout the retina at both 4 and 6 dpf (Fig. 5B, F). When treated with 100 nM T3 from 2 to 4 dpf, *lws1* reporter expression was not altered, but *lws2* reporter expression increased, when endogenous *lws2* normally decreases in response to TH^21^(Fig. 5C). When the thyroid was ablated with Mtz treatment from 2 to 4 dpf and eyes were collected for reporter imaging at 6 dpf, no changes in *lws1* reporter expression were observed, but *lws2* reporter expression showed a significant decrease (Fig. 5G). These results were confirmed with quantification of GFP+ (Fig. 5D, H) and RFP+ (Fig. 5E, I) cells. Overall numbers of reporter positive cells were significantly different between vehicle and T3-treated larvae (*p* = 0.000056; Student’s t-test), which suggests that the increase in RFP+ cells observed may be due to increased expression of RFP reporter in all LWS+ cells, much like the *Δ0.6kb* transgenics. These results suggest that the LRE may be involved in the repression of *lws1* and the normal response to TH at larval timepoints in the in vivo promoter-reporter system.

At juvenile (Fig. 5J) and adult (Fig. 5N) timepoints, however, the pattern of *lws1/2* reporter expression in *ΔLRE* retinas returned almost to normal (similar to the *lws1:GFP-lws2:RFP* transgenic line in juveniles and adults; Fig. 2), with *lws1* reporter expression isolated to the ventral and nasal periphery, and *lws2* reporter expression in the central and dorsal retina. In adults, we observed areas that lacked *lws2* reporter expression in the temporal retina that were not observed in juvenile retinas. When juveniles (Fig. 5K) and adults (Fig. 5O) were treated with 386 nM T4 for 5 days, a response to T4 was observed, with GFP expression throughout the entire retina and RFP isolated to a central region that is completely coexpressed/interspersed with GFP. Juvenile (Fig. 5L) and adult (Fig. 5P) retinas of *ΔLRE* transgenics also displayed typical reporter patterning in response to athyroidy, with *lws1* isolated to a ventral patch, likely due to RA signaling^21,22^. Interestingly, the areas of temporal *lws2* reporter sparseness observed in untreated adult retinas were not found in retinas of athyroid adults. We then quantified the regions of GFP+ only, RFP+ only, GFP/RFP+ coexpressed/interspersed, and no GFP/RFP+ cones as a percentage of the whole retinal area for juveniles (Fig. 5M) and adults (Fig. 5Q). Quantification confirmed the qualitative observations, with the regions of exclusively RFP+ cells decreasing from 73% to 0% in juveniles and 49% to 0% in adults in favor of regions of exclusively GFP + and GFP/RFP interspersion/coexpression in TH treated fish, which increased from 14% to 29% and 13% to 71% respectively in juveniles and 18% to 50% and 9% to 50%, respectively, in adults. Athyroidy had the opposite effect, with areas of exclusively GFP + and GFP/RFP interspersion/coexpression decreasing from 14% to 10% and 13% to 9%, respectively, in juveniles and 18% to 12% and 9% to 0%, respectively, in adults. Areas of exclusively RFP+ cones increased from 73% to 81% in juveniles and 49% to 88% in adults. This suggests that in juveniles, the region within the LRE that was disrupted may not have a role in *lws1/2* patterning or regulation by TH, but in adulthood, the element may be involved in the continued promotion of *lws2* expression in the temporal retina.

We aimed to further investigate the temporal region lacking *lws2* reporter expression in adult *ΔLRE* retinas, so we performed immunocytochemistry on *ΔLRE* adult retinal cryosections using 1D4, which labels both LWS cone opsins in zebrafish^49^(Fig. 5R-R’’), and ZPR1, which labels cone arrestin (Arr3a) present in double cones (all LWS and RH2 cones) in zebrafish^50^(Fig. 5S-S’’). We found that the areas of *lws2* reporter sparseness in the temporal retina were not due to cone death or lack of LWS cones, but due to lack of RFP (*lws2*) reporter expression (Fig. 5R, S). GFP and RFP reporters were coexpressed with both 1D4 (Fig. 5S’,S’’) and ZPR1 (Fig. 5S’,S’’), suggesting correct localization of reporter expression in LWS cones. Overall, the LRE (or the region within that was deleted) may have relatively high importance in the patterning of *lws1/2* and TH regulation early in development, but there may be compensatory mechanisms later in development that allow for the recovery of patterning and TH responsiveness.

## Discussion

**Fig. 6:**
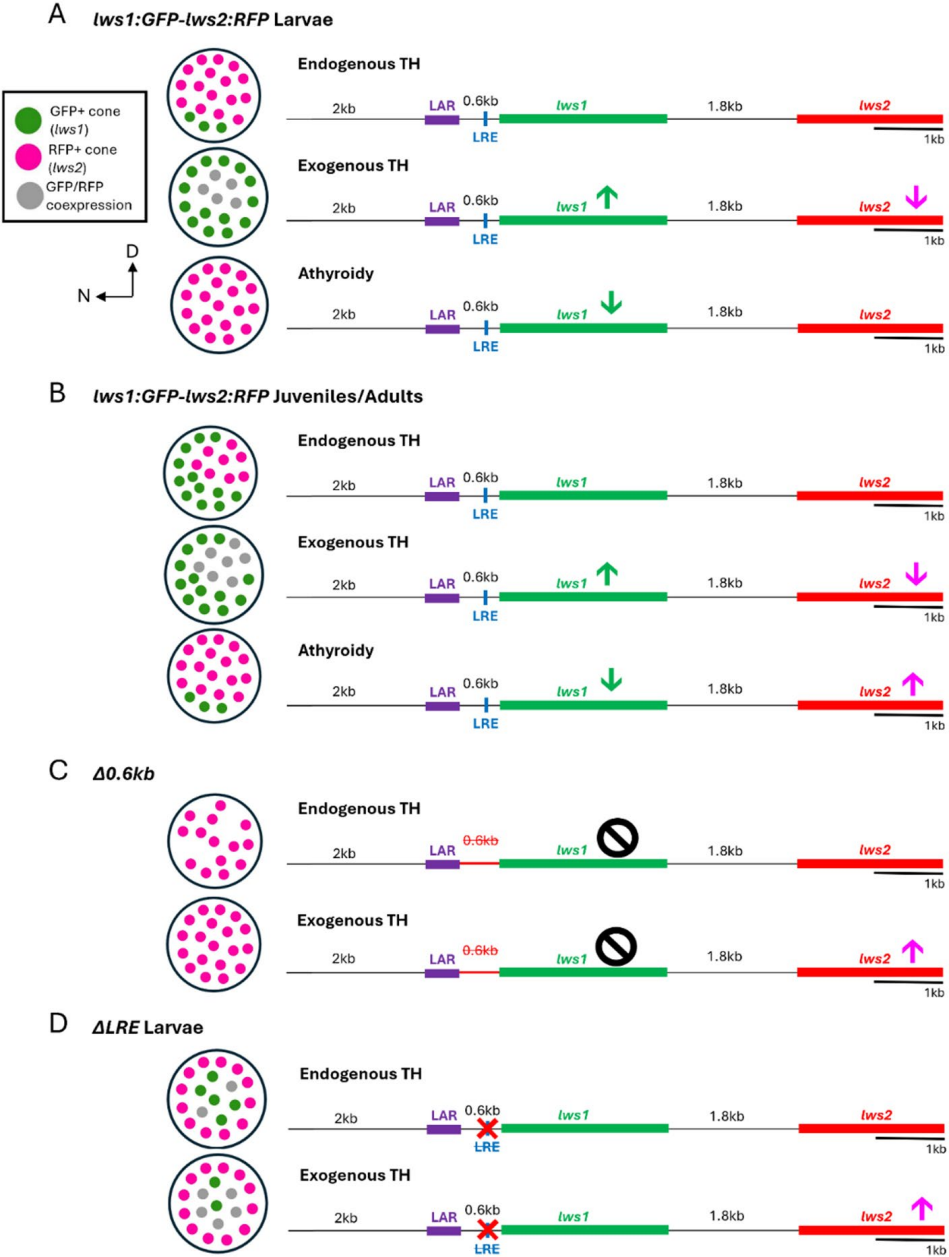
Summary of the potential role of the LRE in zebrafish. **(A)** Diagram depicting the change in the LWS cone mosaic and *lws1/2* reporter expression in the *lws1:GFP-lws2:RFP* transgenic larval zebrafish in response to endogenous TH, exogenous TH, and athyroidy. Reporter expression reflects the normal *lws1/2* cone mosaic and response to TH, with very little *lws1* reporter expressed at endogenous TH. *lws1* reporter expression increases at the expense of *lws2* reporter expression in response to exogenous TH. *lws1* reporter expression decreases in response to athyroidy. **(B)** Diagram depicting the change in cone mosaic and *lws1/2* reporter expression in the *lws1:GFP-lws2:RFP* transgenic juvenile/adult zebrafish in response to endogenous TH, exogenous TH, and athyroidy. Reporter expression reflects the normal *lws1/2* cone mosaic and response to TH, with peripheral ventral and nasal *lws1* reporter expression and central and dorsal *lws2* reporter expression at endogenous TH. *lws1* reporter expression increases at the expense of *lws2* reporter expression in response to exogenous TH. *lws1* reporter expression decreases in response to athyroidy and is isolated to a ventral patch likely due to RA signaling^21,22^. **(C)** Diagram depicting the change in cone mosaic and *lws1/2* reporter expression in the *Δ0.6kb* transgenic zebrafish in response to endogenous and exogenous TH. Reporter expression reflects an abnormal cone mosaic, with *lws1* reporter expression not present and *lws2* reporter expression present throughout the retina. In response to TH, *lws2* reporter expression increases, when it typically decreases in response to TH, and *lws1* reporter is not expressed. **(D)** Diagram depicting the change in cone mosaic and *lws1/2* reporter expression in the *ΔLRE* transgenic larval zebrafish in response to endogenous and exogenous TH. Reporter expression reflects abnormal *lws1/2* cone mosaic and response to TH, with premature ectopic central *lws1* reporter expression, but seemingly typical *lws2* reporter expression at endogenous TH. *lws1* reporter expression is not altered in response to exogenous TH, but *lws2* reporter expression increases. D = dorsal; N = nasal.

Thyroid hormone (TH) has been identified as a crucial nuclear signaling molecule in the regulation of tandemly duplicated opsins in zebrafish^19–21,23^. In this study, we significantly increase the knowledge base on this topic through the identification of a potential regulatory element in the zebrafish *lws* locus. We first demonstrated that the 2.6-kb region upstream of *lws1* is sufficient for the suppression of *lws1* reporter at larval timepoints and the upregulation of *lws1* reporter in response to TH, but the 1.8-kb region upstream of *lws2* is not sufficient for the downregulation of *lws2* reporter in response to TH (Fig. 1). Together, the 2.6-kb region upstream of *lws1* and the 1.8-kb region upstream of *lws2* allowed for the normal expression patterning of *lws1* and *lws2* reporters, and their normal regulation by TH (Figs. 2 and 6A-B). This led us to identify the 2.6-kb region upstream of *lws1* as the most likely location of TH-related regulatory elements in the *lws* locus. To further investigate this area, we found that the 0.6-kb region upstream of *lws1* allows for *lws1* reporter expression and the normal regulation of *lws1/*2 reporters by TH (Figs. 4 and 6C). This led us to target the 0.6-kb region upstream of *lws1* as a likely location of upstream regulatory regions in the *lws* locus.

TH binds to TH receptors (TRs) within cells to exert an effect on gene expression. TRs bind to specific genetic motifs called TH response elements (TREs) to recruit transcription factors^24–26^. TRs can be activators or repressors of genetic expression, and their function is heavily dependent on whether they are liganded or unliganded, and whether they homodimerize with TRs of the same type or heterodimerize with other members of the nuclear hormone receptor family^27–29^. There has yet to be direct evidence that TRs bind at the *lws* locus, but the *TRβ* splice variant, *trβ2*, is expressed in cones and is known to be essential for LWS cone fate and opsin expression^23,39–41,51^. Recent findings in zebrafish have suggested that liganded Trβ2 upregulates *lws1* and unliganded Trβ2 tends to promote *lws2* expression^23^. As we have thoroughly demonstrated that TH acts upon the *lws* locus^19–21,23^, one hypothesis to explain this TH regulation is that TH is acting via TRs that associate with TREs upon the locus. The most direct way to test this hypothesis would be to perform a protein pulldown assay, like chromatin immunoprecipitation (ChIP) sequencing, to determine if nuclear TRs bind at the *lws* locus. However, antibody availability for zebrafish proteins is limited, and there is not currently an antibody targeting zebrafish Trβ2, which would be the most likely candidate. Instead, we used genomic prediction tools^21,43–45^ to identify putative TRE or retinoid X response elements (RXRs) in the 0.6-kb region upstream of *lws1*. We found one such element, the *lws* regulatory element (LRE), which contains one canonical TRE half-site (AGGTCA), a potential inverted repeat-like sequence with a 13-bp spacer, a potential direct repeat-like sequence with a 9-bp spacer, and a DR4-like sequence (Suppl. Figure 1). Though we do not currently have information on which nuclear hormone receptors, if any, bind to the *lws* locus and in what combinations, the LRE element is promising because it has several predicted pairs of binding sites, direct and inverted repeat-like elements. Heterodimers tend to preferentially bind direct repeat elements^29,30^, while homodimers tend to preferentially bind inverted repeat elements^35–38^, so the LRE potentially encompasses receptor combinations of both kinds.

When the LRE was disrupted in a transgenic construct (*ΔLRE*; Figs. 5 and 6D), *lws1* reporter expression was not suppressed at larval timepoints, which suggests a role for the LRE in the restriction of *lws1* expression early in development. In response to exogenous TH treatment, *lws2* reporter expression increased and *lws1* reporter expression was not altered, suggesting the LRE may be a site of TH regulation of the *lws* locus. The increase of *lws2* reporter expression is similar to that observed in *LAR: lws2up1.8 kb: GFP* (Fig. 1) and *Δ0.6kb* (Fig. 4) transgenics. This suggests a potential role for the LRE as a repressor element responsible for depressing *lws2* reporter expression in response to TH, which would also be missing in the *LAR: lws2up1.8 kb: GFP* and *Δ0.6kb* constructs. At later timepoints (juveniles and adults), *lws1/2* reporter patterning and response to TH becomes very similar to that of endogenous *lws1/2*, which suggest that, while the LRE (or specifically the region deleted) appears to be important in normal patterning of *lws1/2* reporters in early development, other mechanisms may compensate for the disrupted LRE later in the zebrafish lifespan. Interestingly, *lws2* reporter expression is absent in the temporal retina of adult *ΔLRE* transgenics but is increased in athyroid adults. This suggests that in adult zebrafish, the LRE may be required for continued expression of *lws2* reporter, or for reporter expression in general from the *lws* locus, but in a spatially-restricted manner. The effect of athyroidy upon this phenotype hints that the LRE may interact with other regulatory elements that also respond to TH. Our genomic prediction analyses carry limitations in that the platforms used to identify putative elements, detected sequences that diverge from those demonstrated to bind TRs, and so we cannot state that the LRE is a TRE or contains TREs (Suppl. Figure 1). With this limitation as a caveat, the prediction analyses also found a second element (predicted palindromic-like repeat with a 1-bp spacer), approximately 0.5 kb upstream of *lws1*^21^ as another candidate for mediating regulation of the locus by TH. Unfortunately, potential founders carrying a promoter-reporter transgene in which this element is disrupted did not survive until adulthood, and we were therefore unable to evaluate this element. Further, because this element is missing from the *LAR: lws2up1.8 kb: GFP* and *Δ0.6kb* transgenics, which each respond to TH by increasing the number of *lws2* reporter+ cells, we believe that other putative TRE(s)^21^, within the LAR or within the intergenic region, may be alternative candidate(s). The LAR is a special case for study since it is an important enhancer^17^. Therefore, testing deletions of the LAR in a promoter-reporter system presents its own challenges. In the future, we aim to uncover more of these elements to further elucidate the genomic regulation of the *lws* locus.

TH and TRs mediate expression of not only zebrafish opsins, but mammalian opsins as well. Blue cone monochromacy is a disease where LWS and MWS cones are absent, and only SWS cones are differentiated. This can be caused by mutations in the *TRβ* gene in humans^52,53^, which suggests that TRβ is necessary for LWS/MWS cone fate. This was later confirmed in *TRβ2* null mice, where only SWS cones formed, not MWS cones (the mouse MWS cone subtype expresses *MWS*, which shares an ancestral *LWS* gene with human *LWS/MWS* and zebrafish *lws1/lws2*)^54^. Therefore, SWS cones may be the default pathway for cone fate, which is inhibited by Trβ2^54^. This has been further confirmed by transcriptomic analysis of human and mouse embryos, where *TRβ2* and *RXRγ* expression is transiently reduced during SWS-cone differentiation during development^55^. RXRγ alone does not induce LWS or MWS cone formation, which suggests it must heterodimerize with TRβ2 to influence cone fate. ChAP-seq and ATAC-seq in mice showed that TRβ2 binds DR4 motifs upstream of the *MWS* opsin gene^42^, which is most often a motif associated with heterodimerization^29,30^. Further, retinoic acid (RA) signaling is known to also regulate expression of *lws1* vs. *lws2*^21,22^, suggesting the potential for cross-talk between the two signaling systems. Since the LRE in zebrafish also contains a DR4-like sequence, it represents an excellent candidate for a Trβ2 heterodimer binding site. Future work utilizing a genetically tagged Trβ2 for protein pull-down assays is required for further confirmation.

## Methods

### Animals

Zebrafish (*Danio rerio*) were bred and maintained in monitored aquatic housing units on recirculating system water at 28.5°C and a 14 h light/10 h dark cycle. Embryos were collected according to^56^, with light onset considered to be 0 h postfertilization (hpf) and embryonic age timed accordingly thereafter, with 24 hpf considered 1 day post fertilization (dpf), 48 hpf considered 2 dpf, and so forth. Embryos/larvae used for whole-mount analyses were kept transparent by incubating them in system water containing 0.003% phenylthiourea (PTU) to inhibit melanin synthesis^56^. We note that this concentration of PTU may interfere with synthesis of T4 when the embryonic thyroid gland has formed, although maternal T3 remains available from the yolk sac^57^. Therefore, analyzed “control” larvae may have slightly reduced endogenous TH. All experiments using animals were performed in accordance with the relevant guidelines and regulations and approved by the University of Idaho’s Institutional Animal Care and Use Committee (IACUC). Methods are also reported in accordance with ARRIVE guidelines. Wild type embryos were of an in-house outbred strain originally obtained from Scientific Hatcheries or Aquatica Tropicals (strain is now available from Segrest Farms, Gibsonton, FL) and are referred to as “wild type” or ”WT.” The two stable transgenic lines *Tg(lws1up2.6 kb: GFP)*#1509^kj17cTg^ *Tg(LAR: lws2up1.8 kb: GFP)*#1499^kj19dTg^ were acquired from the RIKEN Brain Science Institute^17^. *Tg(tg: nVenus-2a-nfnB)*^*wp.rt8*^ was the kind gift of David Parichy^21,46^.

### Cloning of transgenic reporter constructs

The *lws1up2.6-0.6.6 kb: GFP*-*lws2up1.8 kb: RFP*^17^ construct in the pT2GFP-TKPA11 vector was created by removing the *sv40 polyA-lws2up1.8 kb: RFP* from the *lws1up2.6 kb: GFP-lws2up1.8 kb: RFP* construct using NotI and inserting it into the *lws1up2.6-0.6.6 kb: GFP* construct at the NotI site 5’ of the HSV-TK polyA. Correct orientation was confirmed by restriction digestion. The *ΔLRE-lws1:GFP-lws2up1.8 kb: RFP* construct was created by inverse PCR (after restriction digestion and ligation of the template DNA) using primers in Table 1 designed to remove 25-bp of the *lws* regulatory element (LRE) from *lws1up2.6 kb: GFP-lws2up1.8 kb: RFP*. The amplicon was digested with EcoNI to make compatible ends and ligated back together using T4 DNA ligase. Sanger sequencing was used to confirm disruption of LRE element, which revealed a 20-bp deletion within the LRE, which encompassed the canonical half-site, spacer region, and most of the predicted direct repeat half-site (Suppl. Figure 3; primer sequences in Table 1). The GFP used in these constructs is eGFP, and the RFP used is dsRed-Express (BD Biosciences Clontech, Tokyo).

**Table 1:** Primers used for modification of reporter constructs.

Construct	Forward Primer	Reverse Primer
ΔLRE	TGACCTCAACAATTCTTCACCC	CCAACCTCGCTCAGGTAACAGGTAT TGGAAGCAGAAC
PT2GFP TKPApDEST cmcl2:GFP	GGAGATCACTTGGGCCCG GCTGCACAGCACCTTGACCTG	TGTGTGTCGACTGCAGAATTTGATA ATTCACTGGCCGTCG

To facilitate the screening process of identifying transient transgenics, the *cmcl2:GFP* transgenic heart marker was inserted into the *ΔLRE-lws1:GFP-lws2up1.8 kb: RFP* construct by PCR amplification of the *cmcl2:GFP-SV40* element from the pDestTol2CG2 (Tol2 kit #395) vector using the primer set in Table 1 and inserting it between the XhoI and BstBI sites in the multiple cloning site of the pT2GFP-TKPA^17^ vector using the NEBuilder HIFI DNA assembly kit.

### Microinjection and generation of stable transgenic lines

WT embryos were microinjected at the single blastomere stage using 20–30 ng/µL of transgenic construct and 25 ng/µL transposase mRNA. The transposase mRNA was in vitro transcribed from pCS2FA-transposase (TOL2 kit #396) using the mMESSAGE mMACHINE kit (Thermofisher). Resulting mosaic F0 transgenics were crossed with WT. GFP and RFP reporter expression and/or GFP heart marker (when applicable) was assessed to determine germline transmission of transgene. Resulting F1 transgenics were bred with WT to create the F2 generation. F2 generation fish and beyond were used for experiments. A minimum of 3 lines per construct were created and GFP and RFP reporter expression was assessed via confocal imaging of larval whole eyes and adult retinas to ensure lack of insertional effects such as silencing. Resulting transgenic lines include: *Tg(lws1up2.6 kb: GFP)*#1509^kj17cTg^, *Tg(LAR: lws2up1.8 kb: GFP)*#1499^kj19dTg^, *Tg(lws1up2.6 kb: GFP-lws2up1.8 kb: RFP)*^*uoi1500*^, Tg(*lws1up2.6-0.6.6 kb: GFP*-*lws2up1.8 kb: RFP)*^*uoi1501*^, and Tg(*ΔLRE-lws1up2.6 kb: GFPlws2up1.8 kb: RFP)*^*uoi1502*^.

### Thyroid hormone treatments

Treatments in general follow the methods of previous studies^20,21,23,44^. For larval zebrafish, stock solutions of triiodothyronine (T3; Sigma) were prepared in dimethylsulfoxide (DMSO; Sigma) and stored in the dark at −20 °C. At 2 dpf larvae were manually dechorionated and 1000x stock solution was added to the water to result in a final 100 nM concentration (DMSO was at a final concentration of 0.1%). T3, for 48 h of exposure, was selected as the TH treatment for embryos/larvae because T3 accumulates more effectively in embryonic eyes of zebrafish than does T4^58^ and for consistency with prior studies^19–21,23^. Vehicle controls were treated with 0.1% DMSO. Solutions were refreshed every 24 h. For imaging of GFP and RFP reporter expression, 4-dpf or 6-dpf larvae were euthanized by MS-222 immersion and whole larvae were fixed with 4% paraformaldehyde (PFA) in phosphate buffered saline (PBS) overnight at 4 °C. Larvae were then washed twice with PBST for 10 min, once with PBS for 10 min, and then stored in PBS until dissection. Whole eyes were removed and sclera teased away by microdissection. “Coverslip sandwiches” were made by using a 60 × 22 mm coverslip as a base and affixing two 22 × 22 mm coverslips on either end. Whole larval eyes were placed in glycerol between the edges of the smaller coverslips, and another 60 × 22 mm coverslip was affixed overtop. This allows for the imaging of the front and back of each eye without compressing the eye.

For juvenile (1 month post fertilization (mpf); 30 dpf, cannot be sexed at this age) and adult (3–6 mpf; 90–180 dpf; both sexes) zebrafish, stock solutions of thyroxine (T4; Sigma) were prepared in NaOH and stored in the dark at −20 °C. During treatments, zebrafish were maintained individually in 250 mL beakers of system water. 10,000x stock solution was added to the water to result in a final concentration of 386 nM (NaOH final concentration was 0.01% and did not affect system water pH; used as vehicle control because the T4 was prepared in NaOH). This T4 concentration was selected for consistency and comparison of previous work using T4 immersion treatments of post-larval fish^20,21,23,46,59–61^. T4 itself (instead of T3 or a synthetic analog) was used also for consistency with prior work, and with the rationale that immersion of juveniles (or adults) in TH would result in entry via the gills into the bloodstream, and T4 is the main, circulating form of TH^62^. Vehicle controls were treated with 0.01% NaOH. Fish were fed every 24 h and solutions were completely replaced after feeding. Juvenile and adult zebrafish treatments were for a duration of five days. Five days was selected for consistency with prior work using opsin reporter lines^20,21^. No adverse reactions were observed, other than body pigmentation differences as previously reported^20^. For imaging of GFP and RFP reporter expression, fish were euthanized by MS-222 immersion, whole eyes were removed, and retinas were microdissected. Retinas were fixed in 4% PFA in PBS overnight at 4 °C. Retinas were then washed twice in PBST for 10 min, once in PBS for 10 min, and then stored in PBS until dissection. Retinas were whole mounted onto slides and coverslips were affixed using Vectashield Vibrance.

## Thyroid gland ablation

To induce athyroidy, *lws1up2.6 kb: GFP-lws2up1.8 kb: RFP* and *ΔLRE-lws1:GFP-lws2up1.8 kb: RFP* fish were crossed with *Tg(tg: nVenus-2a-nfnB)*^*wp.rt8*^^46^, which expresses the bacterial gene, nitroreductase, in the cells of the thyroid gland via the thyroglobulin promoter. When the prodrug, metronidazole (Mtz), is added to the water from 2.5 to 4.5 dpf, nitroreductase catabolizes it into a cytotoxic compound that selectively ablates the cells of the thyroid. A solution with a final concentration of 10 mM Mtz and 0.1% DMSO was used. 0.1% DMSO alone was used for vehicle controls. Treated larvae were allowed to grow to 6 dpf for larval whole eye collection as described above, or 1 mpf for juvenile and 3–6 mpf for adult whole mounted retina collection as described above. Athyroid zebrafish showed lower survivability during larval stages (14–28 dpf), yielding smaller sample sizes for juvenile and adult experiments. No other negative health effects were observed other than pigmentation differences as previously reported^46^.

### Fluorescence hybridization chain reaction (HCR) in situ hybridization

HCR v3.0 was performed following the manufacturer’s protocol (Molecular Instruments)^63^. After euthanasia by MS-222 immersion, whole 4-dpf larvae were fixed in 4% PFA in PBS overnight at 4 °C, then dehydrated and stored overnight at −20 °C in methanol (MeOH). Before transcript detection, tissues were rehydrated with graded MeOH/PBS/0.1%-Tween washes and post-fixed in 4% PFA in PBS. For hybridization, tissues were incubated in a hybridization oven overnight at 37 °C in a probe solution containing custom-designed, transcript-specific probes from Molecular Instruments (Table 2). Following hybridization, excess probes were removed using the manufacturer’s supplied wash buffers. Tissues were then incubated in amplifier solution at room temperature to allow chain reactions to proceed. Once the HCR reaction was complete, whole larval eyes were removed and sclerae were teased away by microdissection. Eyes were then placed in a coverslip sandwich for imaging.

**Table 2:** NCBI Accession Number for generating HCR probe sets and Molecular Instruments lot numbers.

Gene Name	NCBI Accession Number	Lot Number
*opn1lw1*	NM_001313715.1	RTG197
*opn1lw2*	NM_001002443.2	PRE786
*eGFP*	U55761.1	
*dsRed-Express*	GQ268961.1	

### Immunoctochemistry (ICC)

Whole adult zebrafish eyes were fixed for cryosectioning following previously established protocols^64^. Adult zebrafish were euthanized (anesthetized using MS222 overdose followed by decapitation) and whole eyes were enucleated and lenses were removed. Eyes were fixed in 4% PFA in PBS overnight at 4 °C. Post fixation graded sucrose washes were performed, then stored in 20% sucrose PBS overnight at 4 °C. Eyes were then embedded in 2:1 20% sucrose PBS and OCT medium (Sakura Finetek) and sectioned at 5 μm thickness on a Leica CM3050 cryostat, dehydrated, and stored at −20 °C. Cryosections were thawed at room temperature prior to staining, blocked in ICC blocking buffer for 30 min, and incubated overnight at 4 °C with primary antibody solution. Primary antibodies used include mouse monoclonal ZPR-1 which targets Arrestin3a which is found in double cones (all LWS and RH2 cones) in zebrafish^50^ (1:400; Zebrafish International Resource Center (ZIRC); RRID: AB_10013803 (ZIRC does not supply Lot numbers for their antibodies)) and 1D4 which labels all LWS cones in zebrafish^49^ (1:200; Abcam; Lot 1076899-4, 1096872-9, 1106312-2; RRID: AB_304874). Slides were then washed in PBS with 0.5% Triton-X-100 (PBST) for 30 min before secondary antibody solution consisting of anti-mouse Alexa-Fluor 647-conjugated secondary antibody (1:200; Jackson ImmunoResearch) and DAPI (1:1000) was added and allowed to incubate for 30 min to 1 h. Slides were washed a final time in PBST for 30 min and then coverslips were affixed using Vectashield Vibrance.

### Confocal microscopy and quantification/statistics

Imaging was performed using either a Nikon-Andor or Nikon-Crest spinning disk confocal microscope equipped with a BSI Express 16-bit sCMOS camera. A dry 20x lens was used for larval whole eyes and juvenile and adult whole retinas, while a water immersion 40x lens was used for adult retinal cryosections. A Z-series encompassing the entire eye/retina/section was collected using 2 μm-step z-stacks. Large image stitching was used for whole mounted retinas and retinal cryosections. FIJI (ImageJ) was used to flatten z-stacks via a max projection and adjust brightness/contrast. A Python cell counting script was used in FIJI to count GFP + and RFP+ cones of whole larval eyes. FIJI was used to trace and calculate percent areas of juvenile and adult retinas containing GFP+, RFP+, GFP/RFP coexpressed/interspersed, and no GFP/RFP cones^20,65^. Graphing and statistical analysis were performed using Excel and GraphPad Prism 10.5. Cell quantification data were subjected to a Shapiro-Wilk test for a normal distribution. If, for all conditions, the null hypothesis could be accepted (that the data displayed normal distributions), then statistical comparisons were made using a Student’s *t*-test. If, for any condition the null hypothesis was rejected, then statistical comparisons were made using a Mann-Whitney U test. Regional analysis data were analyzed using the Fisher exact test. Sample sizes (*n*, number of embryos) are provided within Figure legends, along with the p-value and statistical test used in each case.

## Supplementary Information

Below is the link to the electronic supplementary material.

Supplementary Material 1

## Data Availability

All data are contained within the manuscript and supporting information.
