# Supplementary material for: A potential cis-regulatory element regulates differential expression of long wavelength-sensitive opsins in zebrafish
Source: Sci Rep. 2026 Apr 11;16:16965. doi: 10.1038/s41598-026-47667-4 (PMC13230925; doi:10.1038/s41598-026-47667-4)
Supplement: Supplementary file 1 — Supplementary Material 1 [file 41598_2026_47667_MOESM1_ESM.pdf]

Supplemental Figures

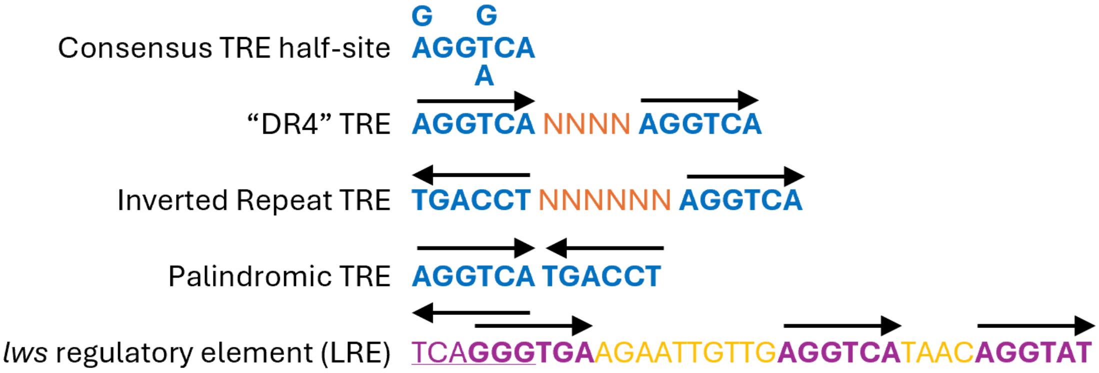

Supplemental Figure 1: Canonical TRE sequences and how they compare to the potential *cis*-regulatory element, the LRE

The sequence of the human canonical TRE half-site and the 3 major human TRE consensus sequences compared to the LRE.

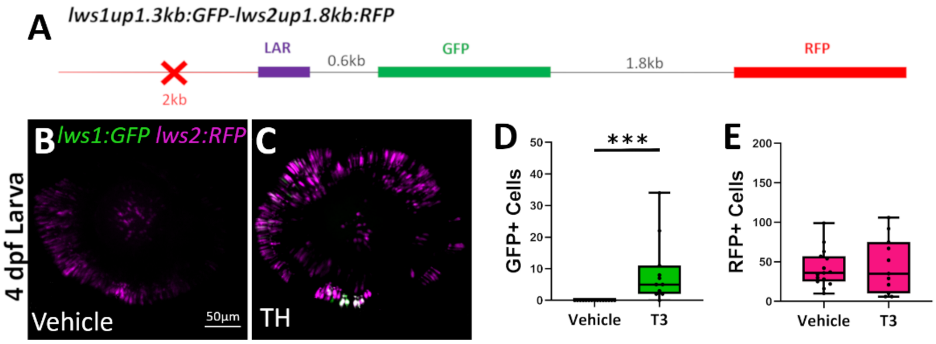

Supplemental Figure 2: The 2-kb region upstream of the LAR does not contain the elements necessary for the endogenous patterning or TH regulation of *lws1/2*

(A) Schematic representations of the *lws1up1.3kb:GFP:lws2up1.8kb:RFP* reporter construct. **(B,C)** Whole-mounted 4-dpf transiently expressing *lws1up1.3kb:GFP:lws2up1.8kb:RFP* eyes visualized using confocal microscopy of **(B)** DMSO-treated (vehicle) or **(C)** T3-treated larvae. **(D,E)** Numbers of **(D)** GFP+ and **(E)** RFP+ cones for DMSO vs T3 treated *lws1up1.3kb:GFP:lws2up1.8kb:RFP* (n = 15 for each; GFP p = 0.00034; RFP p = 0.92). The p values were calculated by comparing GFP+ and RFP+ cones from the treated vs vehicle group using the Mann-Whitney U test; ***$p < 0.001$.

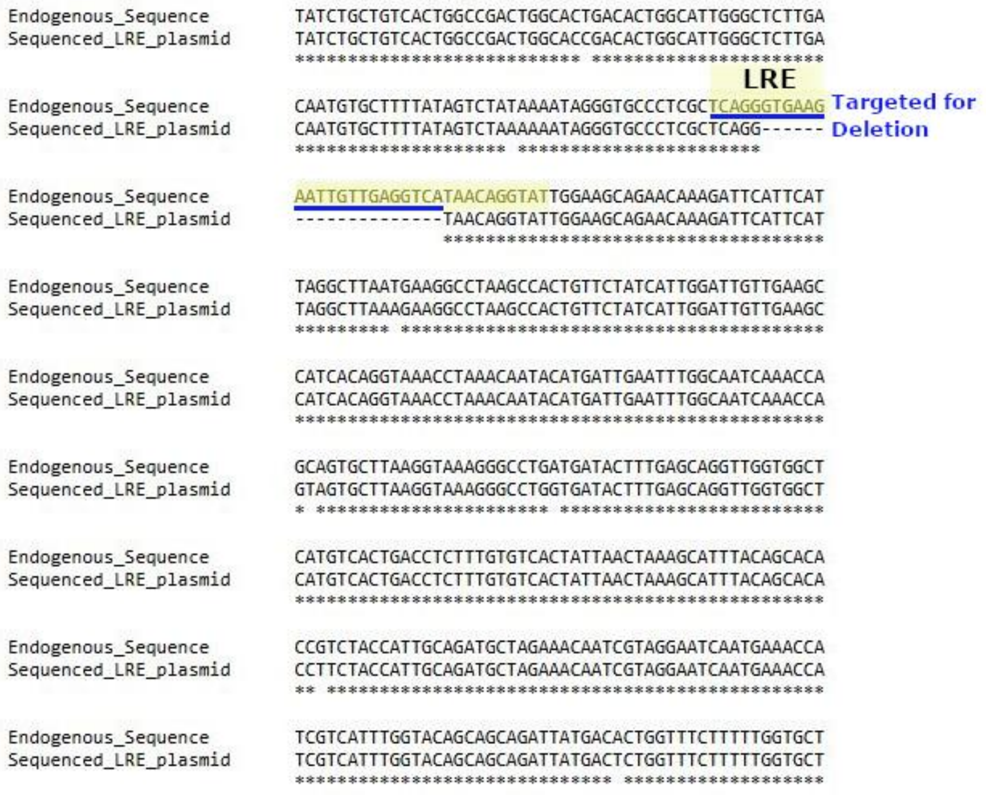

Supplemental Figure 3: The *ΔLRE* reporter construct contains a 20-bp deletion of the LRE

Clustal W alignment of regions of the native sequence ("Endogenous_Sequence") upstream of *lws1* and the sequenced *ΔLRE* plasmid. LRE is highlighted in yellow. Sequence targeted for deletion is underlined in blue. Additional mismatches in this alignment may represent single nucleotide polymorphisms (SNPs), differences between the strain from which the PAC library number 706 was derived (=AB strain) (1) (PAC(H) is the source of all transgenic constructs used in this study, including *ΔLRE*) (2), vs. the reference genome, GRCz11 (=Tübingen strain), used for comparison.

References:

1. Amemiya CT, Zhong TP, Silverman GA, Fishman MC, Zon LI. Zebrafish YAC, BAC, and PAC genomic libraries. Methods Cell Biol. 1999;60:235-58.

2. Tsujimura T, Hosoya T, Kawamura S. A single enhancer regulating the differential expression of duplicated red-sensitive opsin genes in zebrafish. PLoS Genet. 2010;6(12):e1001245.
